# Investing in Public Health Infrastructure to Address the Complexities of Homelessness

**DOI:** 10.3390/ijerph18168887

**Published:** 2021-08-23

**Authors:** John P. Allegrante, David A. Sleet

**Affiliations:** 1 Department of Health and Behavior Studies, Teachers College, Columbia University, New York, NY 10027, USA; 2 Department of Sociomedical Sciences, Mailman School of Public Health, Columbia University, New York, NY 10032, USA; 3 School of Public Health, College of Health and Human Services, San Diego State University, San Diego, CA 92182-4124, USA; davidasleet@gmail.com; 4 Veritas Management Group, Inc., Atlanta, GA 30318-0655, USA

**Keywords:** environment, homelessness, infrastructure, poverty, public health, social determinants of health

## Abstract

Homelessness is now recognized as a significant public health problem in North America and throughout advanced economies of the world. The causes of homelessness are complex but the lack of affordable housing, unemployment, poverty, addiction, and mental illness all contribute to the risk for homelessness. We argue that homelessness is increasingly exacerbated by system-wide infrastructure failures occurring at the municipal, state, and federal government levels and whose catastrophic impacts on population health and the response to the COVID-19 pandemic are the consequence of the decades-long devolution of government and neglect to invest in public infrastructure, including a modern public health system.

## 1. Introduction

The National Homelessness Law Center (formerly the National Law Center on Homelessness and Poverty) has estimated that each year at least 2.5 to 3.5 million Americans are homeless [1]. The homeless, which includes adults and children, are often forced to live and sleep in community shelters, transitional housing, or other urban public spaces that were never intended for human habitation [1]. The National Homelessness Law Center further estimates that, as a consequence of economic necessity, an additional 7.4 million have lost their homes and are living with others because of the vicissitudes of economic cycles. In addition, the U.S. Department of Housing and Urban Development (HUD), in its most recent Annual Homeless Assessment Report to Congress [2], reported that on a single night in January 2020, 580,466 people experienced homelessness in the United States. Compared to 2019, this represents an increase of 2.2% (12,751 homeless people). The report also noted that between 2019 and 2020, homelessness increased significantly among unsheltered populations and those who had been chronically homeless.

Perhaps most strikingly, despite decades of social policy initiatives dating back to U.S. President Lyndon Johnson’s “Great Society” programs that were designed to increase the availability of affordable housing, alleviate shortages, and prevent homelessness, Americans of color continue to be disproportionately represented among the homeless. This is in large part due to racial discrimination, social inequities, and a lack of economic opportunities. Thus, although homelessness continues to be a vexing societal problem, the estimates of homelessness vary considerably due to methodologic variation across studies [3], and some believe that such estimates vastly undercount the true number of people who are homeless at any given time [4].

In this commentary, we argue that homelessness is increasingly exacerbated by system-wide infrastructure failures at the municipal, state, and federal government levels and whose catastrophic impacts on population health and the response to the COVID-19 (SARS-CoV-2) pandemic are the consequence of the decades-long devolution of government pursued by politicians on both sides of the ideological aisle, regulatory obstacles and the lack of political will to invest in public infrastructure, including a modern public health system. The premise underlying our argument is that homelessness is a complex problem that is rooted in multiple structural causes and that several critical elements of national infrastructure—including public health services—should occupy a greater priority in social policy aimed at addressing homelessness.

## 2. The Structural Determinants of Homelessness

According to the National Homelessness Law Center, the key drivers of homelessness include structural determinants such as poverty, a lack of affordable housing, and unemployment [1]. The consequences of homelessness take a considerable toll in terms of human and economic costs to society. Addiction to substances, mental illness, and the lack of access to treatment services for these problems are among the most important consequences with public health impact; but these conditions can also intersect with structural determinants to propel homelessness. Thus, homelessness should be viewed as a complex problem that now occurs increasingly in the context of the failure of municipal, state, and federal governments to invest in a broad range of community infrastructure, including public health.

The public health impact of homelessness is staggering in both the United States and Canada [5,6]. *Community Solutions*, a leading advocacy group whose mission is to eradicate homelessness, believes homelessness now constitutes a major public health crisis [7]. Homelessness has been a chronic public health problem in North America for decades and increasingly can be viewed, in large part, as the consequence of the failure of local, state or provincial and federal governments to make the necessary investments in basic elements of the infrastructure and social policy that would prevent homelessness—more affordable housing, better paying jobs, and more robust public health services.

In addition, the social determinants of health include a broad range of non-medical factors that influence health equity, as well as a wide range of systems and conditions that shape daily life. These include affordable housing, full employment, social and public health services, and others. In addition to physical structures and systems of the built environment (e.g., roadways, bridges, schools, telephone lines, broadband, water and sewage treatment plants, and the power grid), critical “human” and other infrastructure includes educational opportunities, job security, improving working conditions, access to nutritious foods, housing quality, affordable transportation, environmental sanitation, social inclusion, social protection and social justice, and access to quality affordable health services [8]. The problem of homelessness and its impact on society, therefore, is not only deeply rooted, but is also complex and tied to myriad areas of politics, social policy, and the built environment. Consistent and sustained investments in infrastructure in cities, towns, and local communities, thus, could help to address the social determinants of health that are now widely known to be key to improving social circumstances and human health [9].

Take, for example, housing. A large body of research shows that housing leads to better community access to health services and population health outcomes. In one study of health outcomes between the homeless and housed Hispanic population in El Paso, Texas, housing status was significantly associated with access to health care [10]. Despite findings such as these, according to the recent Harvard University Report, The State of the Nation’s Housing 2020 [11], which was sponsored by Habitat for Humanity, persistent unaffordability, growing racial disparities, widespread housing insecurity, and major barriers to home ownership are among the ongoing and formidable challenges we currently face in housing. The decline of government investments in affordable housing, expanding employment opportunities, and reducing poverty that would mitigate such challenges, moreover, has played a contributory role in homelessness. Based on data from the Office of Management and Budget, the Center for Budget and Policy Priorities estimates that federal housing assistance, which includes funding for public housing, Native American housing, and other smaller government programs that are designed to assist communities in providing affordable housing, have remained well below that of the discretionary budget authority for housing assistance that was provided a decade ago [12]. The consequence is that homelessness has increased in both high- and low-income housing markets.

**Figure 1:**
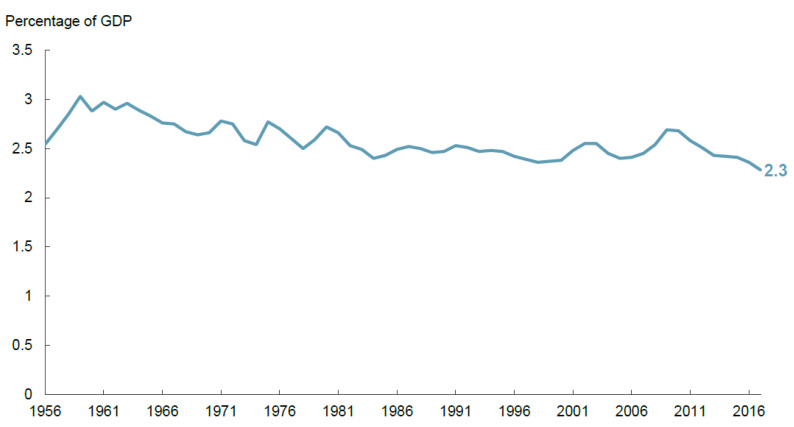
Public spending on transportation and water infrastructure as a share of GDP, 1956–2017. Source: Congressional Budget Office, using data from the Office of Management and Budget, the Census Bureau, and the Bureau of Economic Analysis. GDP = gross domestic product [13].

Similarly, transportation and water—seemingly unrelated to the problem of homelessness—both comprise essential services that play important roles in the health, safety, and productivity of communities. Reliable public transportation infrastructure facilitates people in travelling from home to work and back, and clean water systems ensure the health of communities. Despite the importance of each of these elements of infrastructure, a 2018 Congressional Budget Office analysis of the past 60 years shows that public spending on transportation and water infrastructure as a share of U.S. Gross Domestic Product (GDP), although fairly stable at 2.4 percent, has declined and flattened since 1959, notwithstanding population growth and aging infrastructure (Figure 1) [13]. 

## 3. Infrastructure Failures and their Public Health Impacts 

During the last several decades, Americans have experienced catastrophic failures of infrastructure that have had an increasingly devastating impact on community health and safety across the United States. These failures have threatened the health and safety and either contributed to or exacerbated the problem of homelessness in affected communities. The following are some examples from the last two decades of the more notable infrastructure failures and their impact on public health and safety in the United States:

- In 2005, Hurricane Katrina, a category 5 hurricane, devastated New Orleans, Louisiana, leaving 1800 people dead and estimated thousands more temporarily displaced from their homes and businesses as a consequence of fatal flaws of engineering in the city’s system of levees and the flood protection that the system was supposed to afford homeowners and businesses [14].
- In August of 2007, an eight-lane steel bridge structure that connected Interstate 35 W across the Saint Anthony Falls of the Mississippi River in Minneapolis, Minnesota, collapsed—resulting in the deaths of 13 motorists and injuring over 140 others. According to the National Public Radio, “The bridge collapse sparked immediate calls in Minnesota and across the country [sic] invest big in repairing and replacing the nation’s aging and crumbling infrastructure” [15].
- In one of the most catastrophic failures of state and municipal governments to protect the health of its citizens, the Flint, Michigan, water supply was discovered in 2015 to be contaminated with lead and other toxic chemical wastes, corrosive industrial byproducts, and sewage that were in the Flint River—which became the city’s water supply source when state and local government officials stopped pumping water from a Detroit source in a cost-saving measure and began pumping water from the Flint River into the homes of Flint residents. Since then, one in six of the city’s homes has been abandoned, Flint’s population has plummeted from 125,000 in 2000 to under 100,000 people, and almost half of its residents—most of whom are African American—live below the poverty line [16].
- In February 2021—in the midst of the COVID-19 pandemic—large bands of Texas communities were hit with a power outage that resulted from several severe winter storms that converged and swept across the nation. The outage, which was the consequence of an outdated [17], deregulated and privatized power grid operated by the Electric Reliability Council of Texas (ERCOT) that failed because it had not been weatherized, resulted in severe shortages of food, water, and heat for 4.5 million Texas businesses and homes, and led to the destruction of thousands of homeowner and business properties due to water pipes that froze and burst. The outage killed at least 151 people [18], demonstrating the growing impact of climate change on the built environment.

Each of these examples of infrastructure failure (and countless others that do not make national or international headlines) results in untold consequences for the public’s health and pose grave risks to the most vulnerable populations. Almost all of these calamities resulted in temporary, if not long-lasting, effects on large segments of the population, including traumatic injuries, an increased risk and spread of infectious diseases and housing displacement often leading to temporary or permanent homelessness. More importantly, each of these examples demonstrates the inadequacy not only of the physical infrastructure across the nation, but also the public health infrastructure and preparedness, including facilities and material resources, technology, and the need for a skilled public health workforce to manage emerging public health crises, notwithstanding their explicit root causes.

## 4. The Impact of COVID-19 and the Response of Public Health Systems

The COVID-19 pandemic spawned an increase in visible homelessness due to the COVID-19 social-distancing and other restrictions along with community sweeps, and has only exacerbated the public health consequences for the homeless [19]. In addition to the over 621,000 deaths in the United States and the estimated 4.349 million deaths globally due to COVID-19 (as of 14 August 2021) [20], the pandemic has shown the homeless to be among those at the highest risk for infection, transmission, and death [7]. According to *Community Solutions*:

“the COVID-19 pandemic has exposed the vulnerability of our public health infrastructure and its relationship to housing. During this pandemic, the ability to shelter in place has been key to protecting ourselves and others from the spread of COVID-19. Those who are homeless lack a place to shelter, which puts them not only at higher risk of contracting the coronavirus, but also of spreading it to other vulnerable community members” [7].

The pandemic, thus, further exposed already existing deep fissures and highlighted the deficiencies in the U.S. public health system and preparedness [21,22]. Spending on U.S. public health preparedness has been at issue and a topic of study for more than several decades. One study [23], for example, examined whether changes in spending by local public health agencies over a 13-year period contributed to changes in the rates of community mortality from preventable causes of death. The study found that mortality rates fell between 1.1 percent and 6.9 percent for each 10 percent increase in local public health spending. “These results suggest that increased public health investments can produce measurable improvements in health, especially in low-resource communities” (p. 1585). Additional research [24] using 16 years of data on the relationship between investments in population health and community mortality rates concluded that “incentives and infrastructure supporting multisector population health activities may help close geographic and socioeconomic disparities in population health” (p. 2005). Other studies [25,26] have shown that limiting resources for health-related social services such as housing assistance, employment, access to food, education, and transportation, results in “an anemic infrastructure” for improving population health.

A recent report [27] by the Robert Wood Johnson Foundation and Harvard T.H. Chan School of Public Health, based on a survey conducted between February 11 and March 15, 2021, among a nationally representative, probability-based sample of 1305 adults ages 18 or older, found broad public support for substantially increasing federal spending on public health. According to the survey, about seven in 10 adults (71%) favor substantial new increases in federal spending to improve the nation’s public health programs, while only 27% are opposed. Thus, the COVID-19 pandemic showed Americans that they are once again at a historic inflexion point with regard to funding public health and other elements of public infrastructure that can protect and improve population health.

However, investments in public health would address only the tip of the iceberg. After decades of benign neglect that has resulted in degrading the national public health infrastructure, a key question is whether the United States and other democracies will continue to devolve the government further? Or will the United States and other nations reassert the role and promise of national governments in protecting the health and safety of the public through greater investments in the range of infrastructure projects the United States and other nations need to make to be competitive in the 21st century? 

## 5. Policy Interventions to Improve Infrastructure

In the United States, the Biden Administration’s legislative proposals to invest in roads, bridges, transportation (airports, high-speed rail, electric automobiles), broadband, green and renewable energy, and a modernized national power grid are all laudable and would represent a once-in-a-generation “moon shot” of a national effort to address the nation’s aging infrastructure needs. However, one area of investment that is also critical is the imperative of rebuilding the now chronically underfunded U.S. public health system [28]. Continued failure to invest in our public health system will only contribute to the problem of homelessness and undermine the health needs of those who are experiencing the disruption of a national and global economy in transition. 

In March 2021, the U.S. Congress approved the $1.9 trillion coronavirus relief package authorizing a new Federal spending effort and a temporary, yet dramatic, increase in anti-poverty programs to help millions of American families, including homeless people, who are still struggling amid the COVID-19 pandemic. Under the Biden Administration, U.S. Housing and Urban Development Secretary Marcia L. Fudge [29] unveiled a plan for nearly $5 billion in new grants to states and local governments [30] for rental assistance, the development of affordable housing, and other services to help people experiencing (or on the verge of) homelessness [31]. The grants, which must be spent by 2030, can be used to provide temporary or permanent housing, including buying and converting hotels and motels. Such arrangements could provide the homeless with a more private and safer place to live than in congregate shelters. The grants can also pay for housing for people fleeing domestic violence who have nowhere else to live.

Across the country, however, local governments continue to sweep encampments of the homeless and propose ordinances that would criminalize people who are homeless. In Los Angeles, for example, where state moratoriums on eviction are set to expire in September 2021, the city is now bracing for a surge of people without housing that could fuel the spread of the COVID-19 Delta and other variants [32]. Since March 2020, the Federal Emergency Management Agency (FEMA) has recognized that providing non-congregate shelter for people experiencing homelessness is a powerful public health response to combat the spread of COVID-19. Thus, in response in February 2021, FEMA expanded the available reimbursement for communities providing non-congregate housing.

## 6. Conclusions

An important and growing corpus of scholarship [33,34,35] has emerged on what is being conducted by researchers globally to better understand homelessness, what is now known about its social-structural determinants, and the potential policy and other solutions that can address and mitigate the public health impacts on people who are homeless, including the threat imposed by COVID-19 and its variants. What is clear is that, at a minimum, the most important policy issues that must be addressed to prevent homelessness include housing, an improved economic opportunity and income security, and health [36,37]. In addition, because of the complexity of factors that are involved in understanding the causes and the scope of the potential solutions to homelessness, an applied behavioral and social sciences approach to complex systems will be required if we are to identify and address the challenges in bringing about meaningful and sustainable change in homelessness [38,39].

Apart from research, according to the Harvard Report [11] and Habitat for Humanity, the nation needs to embark on “a comprehensive re-envisioning of national housing policy” to address the structural housing challenges we now face. To do so will require—among other things—confronting the legacy of racial discrimination in housing, creating new sources of government subsidy for affordable housing for low-income households, and designing energy-efficient housing that responds to climate change. It will also require regulatory reform that will remove the significant legal obstacles to building new housing and other infrastructure [40]. However, without a political commitment by governments at all levels to invest in improving infrastructure based on the available evidence of what is needed and what works—specifically, affordable public housing, accessible and affordable transportation, access to clean water and air, and better healthcare and public health services—the homelessness that is rooted in poverty, social inequity and racial discrimination will remain an endemic problem for the foreseeable future in even the most advanced economies.
